# Sequencing the anti-EGFR-TKIs; route to the longest survival

**DOI:** 10.1016/j.esmorw.2024.100074

**Published:** 2024-09-18

**Authors:** H.G. Güzel, Y. İlhan, A.H. Önder

**Affiliations:** 1 Antalya Training and Research Hospital, Department of Medical Oncology, Antalya; 2 Antalya City Hospital, Department of Medical Oncology, Antalya, Turkey

We read the article titled ‘Real-world outcomes of treatment strategy between first-line osimertinib, first/second-generation EGFR-TKIs followed by osimertinib and without osimertinib in advanced EGFR-mutant NSCLC’ by Uehara et al.^1^ Thanks to the authors for broadening the understanding of treatment approaches in epidermal growth factor receptor (EGFR)-mutant non-small-cell lung cancer with real-life data. Nevertheless, we have some concerns to state.

The authors reported that they matched the first-line osimertinib group and the first-line first- (1G) or second-generation (2G) EGFR tyrosine kinase inhibitors (1G/2G EGFR-TKIs) group one by one to carry out a propensity score matching (PSM) based on the FLAURA trial’s^2^ population characteristics to prevent patient selection bias. The results after PSM provide valuable insights. However, the authors split the first-line 1G/2G EGFR-TKIs group into those who could receive second-line osimertinib (second-line osimertinib subgroup) and those who never could. As a result, there was a significant imbalance in the distribution of Eastern Cooperative Oncology Group performance status ≥2 (12% in the first-line osimertinib group and 6% in the second-line osimertinib subgroup) and in the presence of brain metastasis (31% in the first-line osimertinib group and 19% in the second-line osimertinib subgroup). The analyses of the study before the application of PSM, which includes the progression patterns analysis, might have been affected by a patient selection bias.

Another concern is the lack of mutation type (exon 19 del and exon 21 L858R)-based survival analysis. From the results of the FLAURA study, we know that first-line osimertinib did not exhibit an overall survival (OS) advantage over 1G EGFR-TKIs in either the Asian or L858R-mutant subgroups. By contrast, GioTag^3^ and UpSwinG^4^ studies showed better median OS in Asians with exon 19 deletion on sequential treatment with osimertinib followed by afatinib. As the whole population is Asian in this study and both 1G and 2G EGFR-TKIs were used, mutation-based OS analysis might have shed light on this area.

Last but not least, we have doubts about the applicability of the study results in clinical practice. Emerging treatment strategies are being developed for cases of progression on osimertinib. In current practice, these strategies may provide longer survival than what was observed in the retrospective study by Uehara et al., which encourages administering osimertinib. However, half of the patients who do not receive first-line osimertinib may never have the opportunity to benefit from these novel treatment approaches because the acquired T790M resistance after 1G/2G EGFR-TKIs is unpredictable with baseline features. Therefore accurate preselection of the ideal candidates for a non-osimertinib approach is not possible.

In conclusion, this study could provide information to describe patient and disease features that benefit/or not from osimertinib treatment by subgroup comparisons rather than testing the treatment sequence in a population in which we already know osimertinib has a lower efficacy. In addition, choosing a non-osimertinib TKI in the first-line treatment for a convenient patient is like a gamble in today’s practice considering the various post-osimertinib novel treatment strategies and ∼50% ratio of developing T790M mutation. Anyway, we believe that this large study will highlight future perspectives and it is valuable in terms of reflecting the real-world data.
